# Massachusetts Veterinary Association

**Published:** 1891-04

**Authors:** 


*Massachusetts Veterinary Association.*—The regular meeting of the Massachusetts Veterinary Association was held at 19 Boylston Place, Boston, Wednesday evening, January 28th. President Thomas Blackwood in the chair. The Secretary being absent the minutes of the last meeting were not read, and the roll was not called. Dr. J. M. Skally was elected Secretary pro tem. The following members were present : Drs. Blackwood, Osgood,

Marshall, Hadcock, Peterson, Emerson, Lee and Skally. After a general discussion of various topics the meeting adjourned.

J. M. SKALLY, *Secretary pro tem.*

The regular meeting of the Massachusetts Veterinary Association was held at 19 Boylston Place, Boston, Wednesday evening, February 25, 1891. President Thomas Blackwood in the chair. Members present: Drs. Becket, Blackwood, Bunker, Emerson, Hadcock, Marshall, and the Secretary. Visitor, Dr. Wilbert Soule. Minutes of the two previous meetings, November and January read and accepted. The Secretary reported for Dr. Winchester, who was appointed a committee of one to invite Dr. Van Schaick, of the Pasteur Institute of New York, to address the Association upon rabies; that Dr. Van Schaick's time would not permit of his coming to Boston to give such an address at present. There was no essayist for this meeting, but Dr. Bunker agreed to read a paper before the Association at the March meeting, subject to be announced later. The members present than took part in a general discussion upon the following topics:

First, the action of the Cattle Commissioners regarding glanders and tuberculosis, and the folly of three laymen, such as the Board is composed of, in considering themselves as experts on animal diseases, and the harm they do the community in setting the opinion of competent veterinarians at naught. Second, the diagnosis of bovine tuberculosis, especially the difficulty of detecting the disease in its incipiency.

Meeting then adjourned.

AUSTIN PETERS, *Secretary.*

